# Eclipsing Toxicity, Enhancing Efficacy: Clofazimine’s Vanguard in the Realm of Anti-PD-1 and CTLA-4 Immunotherapy

**DOI:** 10.34133/research.0419

**Published:** 2024-07-09

**Authors:** Ruirong Tan, Junning Zhao, Quazi T. H. Shubhra

**Affiliations:** ^1^ Translational Chinese Medicine Key Laboratory of Sichuan Province, Sichuan Institute for Translational Chinese Medicine, Sichuan Academy of Chinese Medicine Sciences, Chengdu 610041, China.; ^2^ National Medical Products Administration (NMPA), Beijing 100038, China.; ^3^ Institute of Chemistry, University of Silesia in Katowice, Szkolna 9, 40-003 Katowice, Poland.

Cancer’s unrelenting grip on global health drives the relentless pursuit of innovative therapies to improve patient outcomes [1]. Among these advancements, immunotherapy—specifically immune checkpoint blockade (ICB)—has emerged as a transformative approach by harnessing the body’s own immune system to effectively target and eradicate tumors [2]. Nivolumab [anti-programmed death receptor-1 (PD1), marketed as Opdivo] and ipilimumab [anti-Cytotoxic T lymp hocyte-associated antigen-4 (CTLA-4), known commercially as Yervoy] are prominent examples of ICB therapy. However, their effectiveness is sometimes overshadowed by severe immune-related adverse events (irAEs) that affect patient quality of life and limit wider application [3]. For example, combined therapy with nivolumab and ipilimumab not only has been shown to lead to higher overall survival rates in patients with cancer with varying programmed cell death-ligand 1 (PD-L1) expression levels but also increases the incidence of grade 3 or 4 irAEs (around 33%) [4].

In advancing ICB therapies, Xue et al. [5] undertook a bold initiative to balance efficacy and safety by screening nearly 3,000 Food and Drug Administration (FDA)-approved drugs, discovering clofazimine (CLF) as a potent enhancer of ICB [5]. Traditionally used for leprosy, CLF now shows promise in cancer treatment, amplifying efficacy and reducing irAEs by enhancing cytotoxic T cell responses and moderating pathogenic T helper 17 (Th17) cell expansion—crucial components given the pivotal role of the adaptive immune system in cancer eradication [6]. Using murine organotypic tumor spheroids from MC38^luc^ colorectal cancers to replicate the tumor microenvironment (TME), CLF synergized with anti-PD-1 + CTLA-4 ICB to substantially improve tumor responses across melanoma, lymphoma, and lung cancer models, and in vivo studies highlighted an 80% tumor-free survival rate in the hypermutated MC38 model, surpassing ICB alone. This success extended to other cancer models like D4M.3A melanoma and A20 lymphoma, where CLF + ICB therapy not only eradicated tumors but also confirmed CLF’s role in augmenting the therapeutic reach of anti-PD-1 + CTLA-4 therapy, marking a significant stride in refining ICB therapy to increase its application and improve tolerability.

The promise of CLF was not only limited to boosting ICB efficacy in eradicating tumors but also uniquely mitigated the severe irAEs often exacerbated by the dual anti-PD-1 + CTLA-4 therapy (Figure) [7]. A focal study on colitis, one of the most challenging irAEs, showed that CLF treatment significantly alleviated symptoms and pathology without affecting the antitumor efficacy of the ICB regimen. CLF notably reduced acute colitis and improved survival in a chronic colitis model where ICB alone led to death within 7 weeks. In models of neurological toxicity, CLF effectively mitigated severe encephalitis symptoms and reversed high mortality rates linked to immune checkpoint therapies. These findings highlight CLF’s dual role in enhancing tumor immunity and protecting against the severe side effects of ICB, supporting a balanced approach to cancer immunotherapy.

**Figure.**
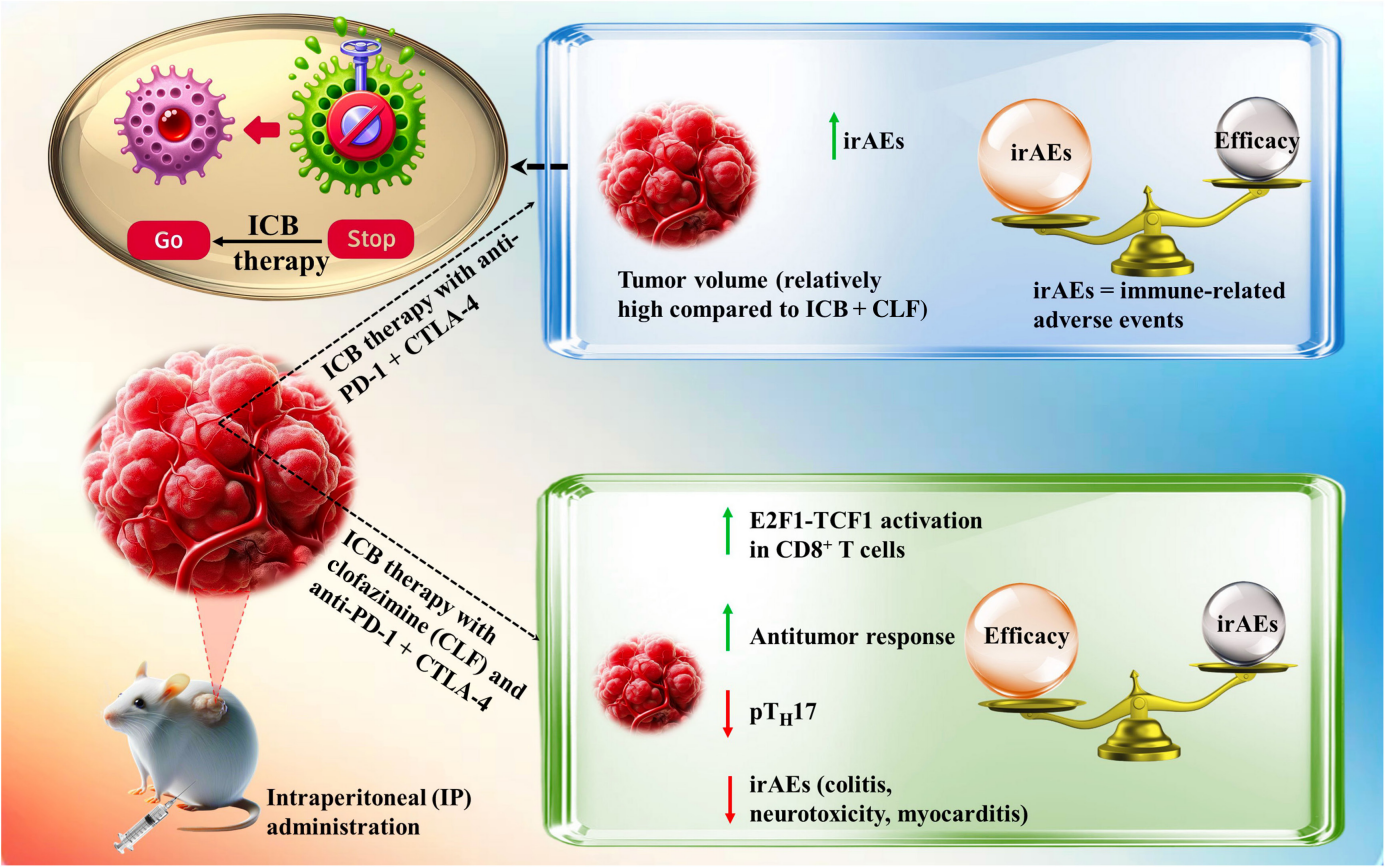
CLF’s role in ICB therapy and irAE modulation. This schematic contrasts the differential outcomes of ICB therapy with and without CLF. The traditional ICB approach leads to relatively less tumor volume reduction and elevated irAEs (up), while CLF coadministration enhances antitumor efficacy and reduces irAEs (down).

Current strategies to manage irAEs from ICB therapy, like reducing ICB dosage or using steroids, have limitations. Reducing the anti-CTLA-4 dose in Xue et al.’s study [5] only modestly improved survival in mice with irAEs, likely due to compromised antitumor effects. While steroids like methylprednisolone can be effective, they often suppress the immune system too broadly, hindering the antitumor response [8]. Xue et al. [5] demonstrate that CLF significantly enhances the efficacy of anti-PD-1 + anti-CTLA-4 therapy, outperforming both dose reduction and steroids in maintaining antitumor responses while also reducing irAEs. While dose reduction lowered irAE mortality from about 45% to 25% to 33% without enhancing survival due to tumor progression, high-dose steroids cut irAE mortality to 33% but impaired antitumor effectiveness. In contrast, CLF reduced irAEs without compromising immune anticancer activity. Notably, CLF has shown exceptional efficacy in mitigating myocarditis, a rare but severe irAE affecting approximately 1% of patients and with a mortality rate over 50%. CLF alleviates myocardial damage—marked by reduced macrophage and T cell infiltration—and counters arrhythmogenic effects, outperforming high-dose methylprednisolone in survival outcomes, confirmed by echocardiographic studies.

Mechanistically, the study demonstrates that CLF enhances the efficacy of dual anti-PD-1 + CTLA-4 ICB by promoting a specialized CD8^+^ T cell population with memory potential, improving tumor eradication across various murine models. Single-cell RNA sequencing revealed that CLF treatment increases M1-like macrophages and reduces exhaustion markers in CD8^+^ T cells, particularly in the CD8_C1 subcluster, which displays higher stem memory and cytotoxic gene expression, such as Tcf7 and Gzmb. This subcluster’s early differentiation state, revealed by trajectory analysis, positions them for effective, sustained antitumor responses. Further mechanistic insights suggest that CLF activates the E2F pathway, enhancing Tcf7 expression and suppressing Pdcd1, which was confirmed by gene set enrichment analysis. Functionally, interferon-γ enzyme-linked immunospot assays showed that CLF boosts tumor-specific cytotoxic responses. Humanized mouse models further demonstrated that CLF not only strengthens antitumor efficacy of nivolumab + ipilimumab but also significantly reduces irAEs, suggesting that CLF could expand the therapeutic window of ICB therapies.

The findings of Xue et al. [5] pave the way for transformative advancements in cancer immunotherapy. This research not only deepens our understanding of how to optimize ICB but also opens new avenues for innovative therapeutic strategies. Mechanistically, the induction of the E2F pathway by CLF—essential for promoting Tcf7 expression and modulating T cell differentiation—emerges as a promising therapeutic target. Leveraging specific E2F agonists or modulators could potentially strengthen and prolong the therapeutic impact of immune responses, thereby sustaining long-term tumor suppression and enhancing T cell memory functionality. Furthermore, CLF’s ability to ameliorate irAEs at a molecular level invites a broader exploration of its interactions within the TME. Investigating how CLF influences various immune cell populations, including natural killer cells, dendritic cells, and myeloid-derived suppressor cells, could yield comprehensive insights into its immunological effects. This could facilitate the application of CLF under other immune-mediated conditions, such as autoimmune disorders or chronic viral infections. The potential of nano-drug delivery systems (DDSs) to improve ICB therapies merits future exploration [9–11]. Smart DDSs engineered for triggered release within the TME and targeted delivery capability hold promise for optimizing CLF + ICB therapy by minimizing side effects. In addition, multifunctional DDSs equipped with imaging agents could facilitate real-time drug tracking and image-guided therapy, enhancing the refinement of CLF + ICB treatment strategies [11].

In conclusion, Xue et al.’s study [5] showcases drug repurposing in cancer immunotherapy, enabling development of more effective and safer ICB therapies through advanced screening and testing.
